# Mapping the network biology of metabolic response to stress in posttraumatic stress disorder and obesity

**DOI:** 10.3389/fpsyg.2022.941019

**Published:** 2022-07-26

**Authors:** Thomas P. Chacko, J. Tory Toole, Spencer Richman, Garry L. Spink, Matthew J. Reinhard, Ryan C. Brewster, Michelle E. Costanzo, Gordon Broderick

**Affiliations:** ^1^Center for Clinical Systems Biology, Rochester General Hospital, Rochester, NY, United States; ^2^Institute of Health Sciences and Technology, Rochester Institute of Technology, Rochester, NY, United States; ^3^Rochester Regional Behavioral Health, Rochester, NY, United States; ^4^War Related Illness and Injury Study Center, United States Department of Veterans Affairs, Washington, DC, United States

**Keywords:** psychoneuroimmunology, metabolism, posttraumatic stress disorder, obesity, computational model, regulatory logic, homeostasis

## Abstract

The co-occurrence of stress-induced posttraumatic stress disorder (PTSD) and obesity is common, particularly among military personnel but the link between these conditions is unclear. Individuals with comorbid PTSD and obesity manifest other physical and psychological problems, which significantly diminish their quality of life. Current understanding of the pathways connecting stress to PTSD and obesity is focused largely on behavioral mediators alone with little consideration of the biological regulatory mechanisms that underlie their co-occurrence. In this work, we leverage prior knowledge to systematically highlight such bio-behavioral mechanisms and inform on the design of confirmatory pilot studies. We use natural language processing (NLP) to extract documented regulatory interactions involved in the metabolic response to stress and its impact on obesity and PTSD from over 8 million peer-reviewed papers. The resulting network describes the propagation of stress to PTSD and obesity through 34 metabolic mediators using 302 documented regulatory interactions supported by over 10,000 citations. Stress jointly affected both conditions through 21 distinct pathways involving only two intermediate metabolic mediators out of a total of 76 available paths through this network. Moreover, oxytocin (OXT), Neuropeptide-Y (NPY), and cortisol supported an almost direct propagation of stress to PTSD and obesity with different net effects. Although stress upregulated both NPY and cortisol, the downstream effects of both markers are reported to relieve PTSD severity but exacerbate obesity. The stress-mediated release of oxytocin, however, was found to concurrently downregulate the severity of both conditions. These findings highlight how a network-informed approach that leverages prior knowledge might be used effectively in identifying key mediators like OXT though experimental verification of signal transmission dynamics through each path will be needed to determine the actual likelihood and extent of each marker’s participation.

## Introduction

The biological and behavioral link between posttraumatic stress disorder (PTSD) and obesity has gathered some empirical traction, particularly because of the elevated co-occurrence of the two conditions (Mitchell et al., 2021). Indeed, veterans with PTSD consistently present with elevated body mass index (Buta et al., 2018) with 83% qualifying as overweight (BMI = 25–29), or obese (BMI ≥ 30) (Hall et al., 2020). This frequency of occurrence does not appear to differ significantly between sexes. A large study conducted recently by the Veterans Health Administration (VHA; Breland et al., 2020, *N* = 4,867,049), found that 44.2% of female veterans had a mean BMI of 29.9 (SD = 6.5) and 41.7% of male veterans had a mean BMI of 29.7 (SD = 5.7), again pointing to an elevated rate of obesity in this population. Importantly, a nationally representative investigation of PTSD and obesity among veterans (*N* = 3,157) found that 5.8% of veterans had co-occurring PTSD and obesity, while 32.7% of them reported obesity and 16.4% reported PTSD (Stefanovics et al., 2020). Moreover, these same authors found that veterans with co-occurring PTSD and obesity also suffered from a higher occurrence of other psychiatric disorders such as anxiety and depressive disorders, suicidal ideation, nicotine dependence as well as a myriad of physical health problems including migraine headaches, diabetes, hypertension, and insomnia.

Current explanations for the convergence of PTSD and obesity often implicate various types of eating disorders [e.g., night eating syndrome (Dorflinger and Masheb, 2018), binge eating (Hoerster et al., 2015), bulimia nervosa (Mitchell et al., 2012), anorexia nervosa (Castellini et al., 2018; Longo et al., 2019), and emotional eating (Dorflinger and Masheb, 2018)], emerging as maladaptive coping strategies, which mediate and maintain the relationship between PTSD and obesity. Consistent with this view, PTSD is widely considered a behavioral risk factor for Metabolic Syndrome (MetS), including diabetes, dyslipidemia, hypertension, and obesity (Bartoli et al., 2013). Indeed, our understanding of the underlying biological regulatory mechanisms that jointly drive PTSD and obesity is still in its nascent stages, particularly as it applies the persistence and co-occurrence of these conditions. In a reductionist approach, investigations of the physiological response mechanisms underlying PTSD have typically focused narrowly on dysregulation of the hypothalamic-pituitary-adrenal (HPA) axis (Dunlop and Wong, 2019). In contrast, a broader physiology has been implicated in obesity, one involving interplay between thyroid regulation, immune function as well as sex hormone regulation (Lainez and Coss, 2019) in maintaining energy homeostasis (Schwartz et al., 2017). Only in recent years have potentially important directions been proposed that focus on the convergence of stress responsive mechanisms active in both conditions (Xiao et al., 2020; Oroian et al., 2021). For instance, Michopoulos et al. (2016) reported on the co-occurrence of PTSD and metabolic disorders such as obesity and diabetes, suggesting that PTSD involves endocrine responses, which might lead to metabolic dysregulation as a function of trauma exposure. More specifically, the latter points to mechanistic interactions linking HPA axis function with that of the hypothalamic-pituitary-thyroid (HPT) axis. Phenotypically distinct yet also co-occurring, PTSD and obesity seem to engage both illness-specific well as overlapping stress-response mechanisms which might be recruited preferentially depending on the individual as well as on the history and context of events to drive the onset of a single or dual pathology.

In this article we leverage the known physiology of HPA, HPT and immune regulation to extract elements of these mechanisms from the peer-reviewed literature using large-scale automated text-mining. Documented interactions linking stress-responsive endocrine and immune mediators are then used to assemble a cohesive mechanistically informed network. We then analyze the uninterrupted paths in the network that facilitate the propagation of stress response through a cascade of metabolic regulatory mediators to affect either pathology independently or both pathologies jointly. By formally representing the close integration of these regulatory axis and the extent of their cross-talk in dictating behavioral responses, such network-informed methods have the potential to be highly effective in identifying key mediators of dysregulation in complex stress-mediated co-morbidities and by doing so offer a new and important perspective on their treatment.

## Materials and methods

### Network creation

In this work we establish an evidence-based regulatory network linking stress, PTSD, and obesity, by extracting endocrine and immune mediators with documented involvement in these conditions as well as their functional interactions from the Elsevier Biology Knowledge Graph database (Elsevier, Amsterdam) (Kamdar et al., 2020) using the Pathway Studio* suite of software tools (Nikitin et al., 2003). Updated on a weekly basis, this database currently recognizes in excess of 1.4 M biological entities (molecules, cell types, diseases, clinical measures, etc.) connected through over 13.5 M relationships (co-expression, regulatory, binding interactions, etc.) extracted by automated text mining from over 5 M full-text peer-reviewed publications and over 32 M PubMed abstracts describing *in vitro* as well as *in vivo* animal and human studies (including results from over 300,000 clinical trials). The query of this database conducted in the context of this work supported an initial 302 regulatory interactions (edges) previously extracted from 10,673 full text of peer-reviewed journal publications using the MedScan natural language processing (NLP) engine (Novichkova et al., 2003; Daraselia et al., 2004).

### Network reliability

Subsequent to model creation, we attempt to control for false positive interactions by testing each of the latter using network analytical concepts proposed by Guimerà and Sales-Pardo (2009) and based on the conservation of connectivity patterns that are broadly conserved in biological and social networks such as modularity or the presence of densely connected subnetworks. By comparing the properties of a given network to those that might be expected in biological networks of comparable size and complexity, the authors compute a reliability *R*_*ij*_ for each network interaction, or the estimated probability that the link “truly” exists given our observation of the whole underlying network. We extend this notion further by the adjusting the probability of an interaction being spurious based on the extent of documentation or number of citations *C*_*ij*_ supporting this interaction. Accordingly, an interaction linking node *i* to node *j* that presents with a high reliability *R*_*ij*_ base on its role in supporting a biologically plausible network structure and that is well documented in addition would correspond to a low spurious score *S*_*ij*_ (Eq. 1).


(1) $$S_{\text{ij}}=\frac{\left(1-R_{\text{ij}}\right)}{C_{\text{ij}}}\;$$

**FIGURE 1:**
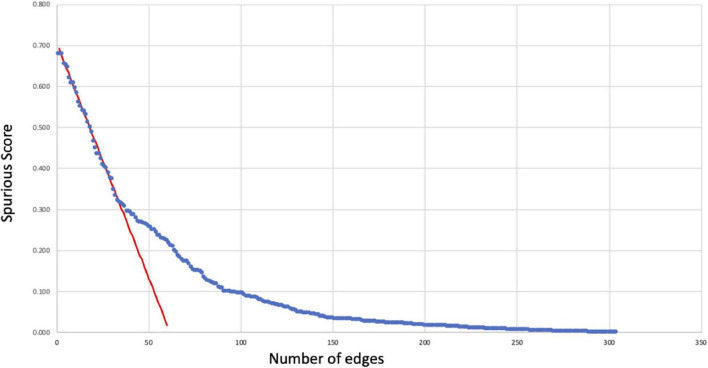
Reliability of regulatory interactions. Spurious scores *S*_*ij*_ (Eq. 1) computed for each of the initial 302 regulatory relationships (blue dots) as an estimate of the likelihood that these interactions might be false positives. These scores decrease linearly (red regression line) to *S*_*ij*_∼30% below which the rate of change slows considerably. As a result, regulatory relationships with *Sij* ≥ 30% were removed as having a high likelihood of being spurious or false positive occurrences.

The spurious score *S*_*ij*_ was computed for each of the initial 302 regulatory interactions producing values ranging from 0 to roughly 68% that were in turn used to prune the network. We observe that these values decrease almost linearly for the 50 or so highest values. As shown in Figure 1, a linear regression was fit to the decrease in spurious score in this region (observations 10 through 30) producing an adjusted *R*^2^ value of 0.98 with an average absolute residual error of 0.007. Using a standard one-sample *t* test to identify significant deviations from this linear regression we found that these occurred consistently with *p* < 0.01 for spurious scores below 30% indicating an inflection point and significant stabilization in spurious score. Accordingly, associations with spurious scores above this threshold were considered unreliable and removed from the final network (see Supplementary Table 1).

### Analysis of network structure and traversing paths

As a means of assessing the degree of biological fidelity capture by this literature-informed network we computed various fundamental network topological features and properties (Barabási and Oltvai, 2004; Huber et al., 2007; Mason and Verwoerd, 2007). These include measures describing the general structure of the overall network such as the as its size and complexity such as network diameter and the network connection density. The network diameter represents the breadth of the network and is computed as the shortest distance between two most remote nodes. Likewise, we computed the characteristic path length, or the mean minimal distance between any two nodes, as a measure of the efficiency of information propagation through the network. As an indicator of network complexity, we computed the network connection density, or the total number of edges in the current network represented as a fraction of all the possible edges in a fully connected network with the same number of nodes. This measure is known to vary significantly across levels of biology and physiological compartments (Frankenstein et al., 2006). Finally, as connection patterns in biological networks tend to favor the emergence of highly subnetworks, or clusters, we also compute the network clustering coefficient (Guimerà and Sales-Pardo, 2009; Di Camillo et al., 2012) using the software package Cytoscape (Shannon et al., 2003).

At the level of individual nodes, we computed different centrality measures to describe their relative role within the network. First, we estimated the closeness centrality to describe how well-connected a given node is to the remainder of the network overall. This is computed as the average length of the shortest path between a given node and all other nodes in the network. To describe how a node might act as a key broker of information or gatekeeper between adjacent highly connected sub-networks, we computed the betweenness centrality. The measure is proportional to the frequency with which a node is positioned along the shortest paths between two other nodes. This same concept is extended to a more detailed analysis of minimal path length where we computed all paths of minimal length containing one or two intermediate nodes separating stress from obesity and stress from PTSD. These network analyses were conducted using the Python package NetworkX (Hagberg et al., 2008).

## Results

### General characteristics of the network

**FIGURE 2:**
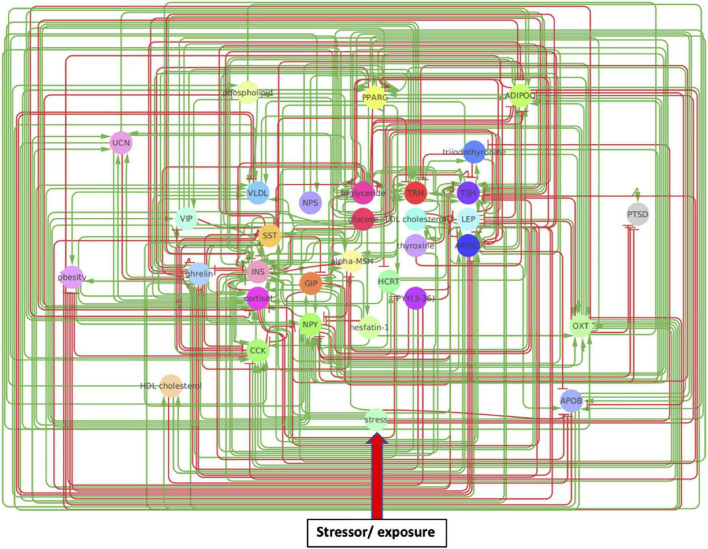
A metabolic network model. Created through Pathway Studios, this model involves directional effects between metabolic markers implicated in PTSD and obesity. Arrows indicate directional regulatory edges between mechanisms, such that a green arrow indicates the source node upregulates the target node while a red arrow indicates the source node downregulates the target node.

The original network produced by the NLP text-mining consisted of 33 nodes and 302 regulatory interactions. Analysis of the estimated reliability if these interactions indicated that roughly 1 in 8 of these (∼34 edges) did not contribute toward improving alignment of the overall network topology with what might be expected of a typical biological network containing the same number of nodes. These were removed to yield a truncated network consisting of the same 33 nodes connected through 268 regulatory interactions supported by a total of 10,637 reference citations (Figure 2 and Supplementary Table 1A). The functional class assigned to these source-target relationships by MedScan (Novichkova et al., 2003; Daraselia et al., 2004) included direct regulation (23 edges), regulation (44 edges), molecular synthesis (19 edges), molecular transport (122 edges), and co-expression (60 edges). Relationships where the mode of action was not assigned were excluded leaving 179 relationships where the source upregulates the downstream target (positive polarity) and 89 where it downregulates the latter (negative polarity). On average, each of the 33 nodes interacted through these functional relationships with approximately 8 upstream and 8 downstream neighbors on average (Supplementary Table 1B) leading to an overall network connection density of roughly 25%. Perhaps not surprisingly, insulin (INS) and glucose appear as highly influential nodes with insulin having the highest number of upstream regulators (indegree 24) and glucose affecting the largest number of downstream targets (outdegree 20). High closeness centralities for both nodes (0.67 for insulin and 0.76 for glucose) indicated that these connections were distributed in a way that support a broad network-wide involvement. Insulin also emerged as the dominant mediator of information flow across the various network neighborhoods with a betweenness centrality of 1.88, or roughly 50% more than its closest rivals triglyceride energy stores (betweenness 1.16), TSH (betweenness 1.06), and ADIPOQ (betweenness 1.01).

While a dominant role of mediators closely related to the regulation of glucose makes intuitive sense and would support the plausibility of this metabolic response network it is also important to examine overall network structure and patterns of information flow (Supplementary Table 2). With respect to the overall breadth of the network, the shortest path linking the 2 most remotes nodes consisted of 4 cascading relationships (network diameter of 4) with on average any pair of nodes being separated by at least two edges or at least one intermediate mediator node (1.87 characteristic path length). On average nodes in the immediate neighborhood of any given node in this network would connect would connect with each other with a connection density exceeding 40% of all possible neighborhood interactions (0.41 clustering coefficient). A cursory examination of similar statistics reported in the literature describing biological networks existing at different levels of granularity suggest that the metabolic response network presented here shares many structural similarities with organ-level networks as opposed to intra-cellular or social networks (Albert and Barabási, 2002; Supplementary Table 2). Indeed, a map connecting 55 functional regions of whole cerebral cortex in cat (Hilgetag et al., 2000) share a strikingly similarity with the network presented here in terms of overall connection density, average number neighboring nodes and characteristic path length separating nodes. Likewise, a theoretical network based on the functional connectivity in cat and macaque monkey cortex (Young, 1993; Sporns and Kötter, 2004) and enriched in functional motifs delivers a virtually identical clustering coefficient of approximately 0.4. These similarities with functional connectivity in mammalian brain contrast sharply with the much lower clustering that appears characteristic of neuronal networks in *C. elegans* (0.28) (Watts and Strogatz, 1998) and the much higher clustering in social interaction networks (e.g., film actors) (Watts and Strogatz, 1998) and language (Yook et al., 2001). Similarly, while intracellular metabolic pathways exhibit a network diameter of similar magnitude, individual nodes are only half as connected with neighbors on average (Jeong et al., 2000). Though not an exhaustive comparison, the dominant role of core glucose regulators as well as the overall topological organization of the propose text-mined response network would suggest that it is to a large extent compatible with known functional networks existing at a similar level of biology.

### Shared and unique mediators of posttraumatic stress disorder and obesity

**FIGURE 3:**
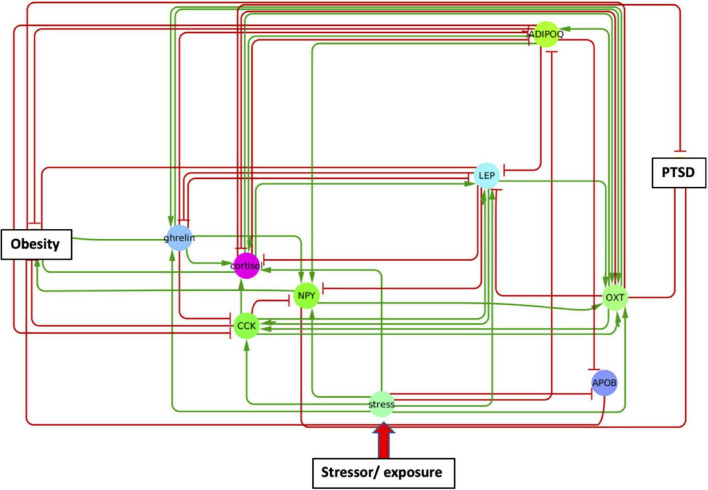
A single metabolic mediator subnetwork. Subnetwork of shortest paths linking stress to PTSD and obesity through only one intermediate metabolic mediator. Shared paths leading to both PTSD and obesity involved the convergent actions of cortisol, oxytocin (OXT), and neuropeptide Y (NPY).

**FIGURE 4:**
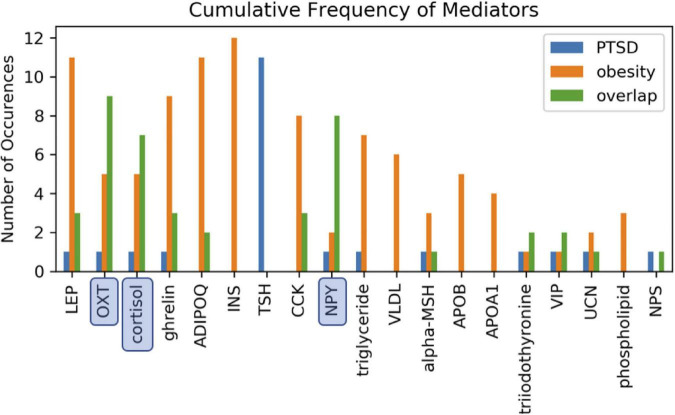
Involvement of cascading regulators. Mediation of one or both health conditions involving two-intermediate metabolic regulators reported as the number of available path occurences. Paths annotated as overlapping involve the same two intermediate mediators to simultaneously link stress to both PTSD and obesity. Oxytocin (OXT), neuropeptide Y (NPY), and cortisol were most frequently involved in jointly mediating PTSD and obesity.

In order to identify metabolic mediators that play a key role in exacerbating PTSD and obesity, we conducted an analysis of the shortest possible paths facilitating the transmission of stress response onto these two pathologies. We found 11 such direct paths where stress affected these pathologies directly by regulating only one of eight possible intermediate network nodes. These consisted of adiponectin (ADIPOQ), apolipoprotein B (APOB), the gastrointestinal hormone cholecystokinin (CCK), cortisol, ghrelin, leptin (LEP), neuropeptide Y (NPY), and oxytocin (OXT). While five of these exercised a direct and unique effect on obesity only, both pathologies were jointly affected by the remaining three, namely cortisol, NPY, and OXT (Figure 3 and Supplementary Table 3). Unfortunately, cortisol and NPY while mediating reduced severity in PTSD are predicted to concurrently exacerbate obesity. Indeed, only increased levels of OXT expressed in response to stress were predicted to jointly reduce severity of symptoms in both PTSD and obesity. Interestingly this effect continues to apply to stress response involving a cascade of two intermediate mediators (Supplementary Table 4A). We found a total of 76 such two-step regulatory cascades. Of these 21 jointly propagated the effects of stress onto both pathologies concurrently. Of these only seven concurrently alleviated the severity of PTSD and obesity. All involved transmission of stress response through OXT via one of the following upstream regulators: CCK, cortisol, ghrelin, LEP, fear mediator Neuropeptide S (NPS), NPY or through vasoactive intestinal polypeptide (VIP). This being said, transmission of stress through regulation of OXT simultaneously exacerbated both pathologies when also mediated through thyroid hormone triiodothyronine or T3. All other shortest stress response paths promoted divergent effects, alleviating one pathology while exacerbating the other. In addition to these stress response cascades jointly affecting both pathologies, we identified 45 paths involving two sequential mediators that uniquely affected obesity (Supplementary Table 4B), 25 of which promoted a stress-induced reduction in obesity. Likewise, we identified 11 cascades through which stress uniquely affected PTSD and not obesity (Supplementary Table 4C). Interestingly, thyroid-stimulating hormone or TSH is common to all these metabolic pathways. Four such pathways attenuated the effects of stress and reduced PTSD severity. These involved upstream regulation of TSH by either cortisol, ghrelin, NPY or OXT. The frequency with which all of the individual network elements mentioned above are recruited into these stress response pathways is summarized in Figure 4.

## Discussion

Posttraumatic stress disorder and obesity are two major prevalent public health concerns in the United States across both military veterans and the general public (Farr et al., 2014). Given the pervasiveness of these two conditions and the inadequate understanding of their underlying mechanisms and pathways, the current study explored how stress affects PTSD and obesity through varied metabolic mediators. We find multiple regulatory cascades involving as few as one or two mediators that support the mechanistic engagement of distinct metabolic response processes to stress that jointly affect PTSD severity and obesity. Whereas many of these paths drove the severity of PTSD and obesity in opposing directions, stress-mediated release of oxytocin was found to concurrently downregulate the severity of both conditions. These results suggest that established graph theoretical concepts might be applied to existing peer-reviewed knowledge to discover the basic physiological mechanisms recruited in support of this co-morbidity.

First and perhaps foremost, it is interesting to observe that the current body of published knowledge contained sufficient information of individual elemental interactions to derive a regulatory network linking an environmental trigger such as stress with health outcomes, both physiological and behavioral, through known metabolic circuitry. Not only was this text-mined network cohesive, allowing for continuous directed paths to each pathology, but it displayed topological features and information flow patterns that were consistent with those broadly conserved across biological networks (Guimerà and Sales-Pardo, 2009; Di Camillo et al., 2012). Moreover, the architecture of this network was especially consistent with real-world functional networks reported in mammalian brains (Albert and Barabási, 2002), a level of physiology particularly relevant to this work. Of note, almost one third of the cascades theoretically available to support the propagation of stress response across this model network jointly affected both pathologies, a proportion reminiscent of the ratio of PTSD and obesity comorbidity reported by Stefanovics et al. (2020). NPY, cortisol, and OXT played a key role in directly propagated stress response to PTSD and obesity as well as in cascades recruiting other metabolic mediators. Interestingly, with the exception of the thyroid hormone T3, all cascades involving OXT were predicted to concurrently alleviate the effects of stress on PTSD severity and obesity. In contrast, NPY and cortisol were predicted to exert divergent effects on these health outcomes. Low levels of NPY are found to contribute to chronic PTSD (Rasmusson et al., 2000; Sah et al., 2009; Sah and Geracioti, 2013; Tural and Iosifescu, 2020) and the efficacy of NPY administered intranasally toward relieving PTSD symptoms is supported in empirical studies (Sayed et al., 2018) earning it the name “resiliency hormone” (Sah et al., 2009). Unfortunately, there is an even larger body of evidence indicating that NPY, a potent orexigenic (appetite-inducing) peptide (Beck, 2006; Assan et al., 2021), when released in response to stress stimulates adipogenesis, inducing an increase in adiposity and exacerbating obesity as well as triggering a cascade of other metabolic alterations (Masodkar et al., 2016; Ailanen et al., 2017). The involvement of cortisol highlights the metabolic sequalae to stress. Once again, the network model presented here predicts that this HPA regulated glucocorticoid will drive divergent outcomes in these pathologies, with stress-induced cortisol exacerbating obesity, while reducing PTSD severity. Expressed at lower basal levels resulting from a maladaptation of the HPA axis to increased GR sensitivity, the role of cortisol in PTSD has justifiably been broadly studied (Dunlop and Wong, 2019), though much less so in obesity (Oroian et al., 2021). Nonetheless, the exogenous role of stress impacting obesity through elevated cortisol reactivity and maladaptive coping behaviors (e.g., comfort eating) has gained much empirical traction (Herhaus et al., 2020). Indeed, heightened consumptive behaviors as a function of HPA axis reactivity was found when CRH was endogenously administered to healthy non-obese adults (George et al., 2010).

The joint mediation by the oxytocinergic system across PTSD and obesity is further described in focused work by Thienel et al. (2016) as well as that of Witteveen et al. (2020). Broadly considered a “natural medicine” attenuating the effects of stress, and promoting resilience and healing (Carter et al., 2020), OXT has been found to impart both preventive and curative effects in PTSD (van Zuiden et al., 2017; Donadon et al., 2018). Importantly, a hybrid intervention with OXT and psychotherapy has been shown to have a potent effect on PTSD severity (Flanagan et al., 2018), though these effects reportedly vary across the sexes (Frijling et al., 2015). Likewise, OXT, being anorectic, functions as a “nutrient status sensor” (McCormack et al., 2020, p. 122), and is found to reduce obesity across humans, rodents, and non-human primates (Niu et al., 2021), through its effect on consumptive behaviors by simultaneously regulating cognitive control as well as food reward processing (Spetter et al., 2018). Importantly, increased OXT signaling and secretion are known to regulate both energy intake and energy expenditure which result in weight loss by its effect on fat mass loss (as opposed to lean mass loss) (McCormack et al., 2020). In addition, there is evidence that OXT is involved in reversing insulin resistance and glucose intolerance, thus reducing obesity (Zhang et al., 2013). Collectively, these results suggest that OXT, an essential regulator of the gut-brain axis (Olszewski et al., 2017) also exercising key roles in various other neurobehavioral pathways and homeostatic systems (McCormack et al., 2020), might be a biomarker and even a potential target of intervention worthy of further study in the management of both PTSD and obesity.

Though not an exhaustive validation of the over 260 regulatory interactions captured in this network, interpretations of the literature made by the MedScan natural language processing engine would appear consistent with those of the human reader, at least in this focused verification those metabolic mediators identified as playing a key role in the comorbidity of obesity and PTSD. This technology continues to evolve and though results are highly dependent on the validation set and metrics, it is not unreasonable to expect a disambiguation accuracy exceeding 85% (Yepes and Berlanga, 2015) in the interpretation of medical texts. This is consistent with the experience of our group where we found accuracy of interpretation exceeding 90% in an endocrine regulatory network counting over 200 interactions validated by consensus with a second NLP engine and a human domain expert reader (Morris et al., 2019). It is also important to remember that in this work every citation was attributed an equal credibility in its support of a given interaction, irrespective of the possible differences in publication date or source publication. The development of useful quality metrics continues to be an active field for our group (Jackson, 2021) and others. Although they are useful in highlighting those regulatory cascades through which stress propagate, it is important to remember that these paths were identified based on connectivity alone. Though theoretically available, the relative kinetics of signal transmission through each path will determine the actual likelihood and extent of its participation. Nonetheless we propose that the current analysis offers an efficient framework for collecting, reconciling, and operationalizing existing community knowledge toward informing on key markers in the design of studies that can precede even pilot level investigation. Moreover, the resulting networks offer a mechanistically informed description of biology relevant to an illness of interest without being illness-specific. Hence, representing accrued knowledge in this way creates a lasting and widely applicable model of what we know that transcends a specific study data set while also ensuring the consistency of the latter with peer-reviewed observations of the broader research community.

## Data availability statement

The original contributions presented in this study are included in the article/Supplementary Material, further inquiries can be directed to the corresponding author.

## Author contributions

TPC was primarily responsible for literature review, model creation, results analysis, and manuscript writing. SR was primarily responsible for network analysis and generation and interpretation of results. JT was primarily responsible for supervision of manuscript writing, expertise on computational modeling techniques, and clinical applications. GLS, MJR, RCB, and MEC contributed to the design of the study as well as the interpretation of results, assessment of clinical validity, and writing of the manuscript. GB was responsible for oversight and funding of project, definition and supervision of the research, manuscript writing, and expertise on computation modeling techniques. All authors contributed to the article and approved the submitted version.

## Author disclaimer

The opinions and assertions contained herein are the private views of the authors and are not to be construed as official or as reflecting the views of the Department of Defense, Rochester Regional Health, or Elsevier BV. Pathway Studio is a trademark of Elsevier Limited. Pathway Studio is copyright © 2020 Elsevier Limited except certain content provided by third parties.
